# Epigenetic inheritance and the missing heritability

**DOI:** 10.1186/s40246-015-0041-3

**Published:** 2015-07-28

**Authors:** Marco Trerotola, Valeria Relli, Pasquale Simeone, Saverio Alberti

**Affiliations:** Unit of Cancer Pathology, CeSI, Foundation University ‘G. d’Annunzio’, Chieti, Italy; Department of Neuroscience, Imaging and Clinical Sciences, Unit of Physiology and Physiopathology, ‘G. d’Annunzio’ University, Chieti, Italy

**Keywords:** DNA methylation, Missing heredity, Transgenerational inheritance

## Abstract

Genome-wide association studies of complex physiological traits and diseases consistently found that associated genetic factors, such as allelic polymorphisms or DNA mutations, only explained a minority of the expected heritable fraction. This discrepancy is known as “missing heritability”, and its underlying factors and molecular mechanisms are not established. Epigenetic programs may account for a significant fraction of the “missing heritability.” Epigenetic modifications, such as DNA methylation and chromatin assembly states, reflect the high plasticity of the genome and contribute to stably alter gene expression without modifying genomic DNA sequences. Consistent components of complex traits, such as those linked to human stature/height, fertility, and food metabolism or to hereditary defects, have been shown to respond to environmental or nutritional condition and to be epigenetically inherited. The knowledge acquired from epigenetic genome reprogramming during development, stem cell differentiation/de-differentiation, and model organisms is today shedding light on the mechanisms of (a) mitotic inheritance of epigenetic traits from cell to cell, (b) meiotic epigenetic inheritance from generation to generation, and (c) true transgenerational inheritance. Such mechanisms have been shown to include incomplete erasure of DNA methylation, parental effects, transmission of distinct RNA types (mRNA, non-coding RNA, miRNA, siRNA, piRNA), and persistence of subsets of histone marks.

## Introduction

Patterns of heritable traits within the human population determine body phenotypes, through a deeply intertwined interaction between genetic components and the environment. Specific genetic/DNA sequence variants are typically inherited transgenerationally as Mendelian alleles and are supposed to carry with them all the genetic information that acts as inheritable determinant [1]. Genome-wide association studies (GWAS) have recently demonstrated that multiple genomic loci are linked to complex traits, such as body development and height ([2, 3] and references therein). Several common disorders, such as type 2 diabetes, Crohn’s disease, and rheumatoid arthritis, were also shown to possess significant genetic components, as provided by multiple polymorphic loci [4–6]. These findings led to postulate models whereby numerous genetic factors provide small, independent contributions to complex phenotypes, such effects being essentially additive [5]. However, simple models of additive effects of ever-smaller components have remained as yet unproven [4–7]. On the other hand, identified genetic factors associated with complex diseases have been found to confer far less disease risk than expected from empirical estimates of heritability and typically explain only a minority of the heritable traits. As a consequence, “pure” genetic models are prone to underestimate the interactions among loci [5], globally designated as epistasis. Epistatic components need to be integrated by estimates of the contribution of non-genetic factors, globally designated as the “missing heritability” [7, 8]. Hence, the issue remained open, whereby identified genetic factors associated with complex diseases conferred far less disease risk than expected from empirical estimates of heritability. As an example, Crohn’s disease is a recessive disorder which shows about 80 % heritability. However, the genetic components identified to date only explain 20 % of this heritable fraction [9]. An additional example is that of human population stature [2, 3]. A significant fraction of height-determining genes has been identified by GWAS analysis [10–12]. Most of these genes were demonstrated to be largely overlapping in Caucasian and non-Caucasian populations [13, 14], consistent with an actual identification of the most relevant height-associated determinants. However, the identified polymorphisms were found to account only for a minor fraction of stature heritability. Although dedicated procedures for SNP-associated analyses have significantly increased their combined predictive power [15], a large amount of heritable height-associated factors remains undetectable by conventional GWAS, suggesting that such non-DNA sequence-linked information may be associated to epigenetic heredity.

### Epigenetic heritability

Epigenetic modifications, such as DNA methylation, can contribute to alter gene expression in heritable manner without affecting the underlying genomic sequences. Such epigenetic contribution would be systematically missed by conventional DNA sequence-based analyses. A model of epigenetic inheritance, as additional to Mendelian heredity of polymorphic DNA sequences, would thus efficiently explain the lack of detection in conventional GWAS as “missing heritability”. It would also help explaining the cases of rapid, heritable adaptations to changing environmental conditions, such as for human stature [2, 3], and the occurrence of hereditary epistatic effects. Support for this model is provided by the evidence that phenotypic plasticity can emerge over rapid time scales, at rates that are orders of magnitude higher than the processes of natural selection [16, 17].

However, to be tenable, such a model of epigenetic inheritance poses rigorous requirements: (a) mitotic inheritance of epigenetic traits across cell generations (see discussion on DNA methylation maintenance through mitotic cycles); (b) epigenetic inheritance across successive meiotic divisions (see the paragraphs describing gamete generation and the development of primordial germ cells (PGC); and (c) true transgenerational inheritance, which requires proof of heritability beyond the first generation that has not been unexposed to the causal epigenetic modifiers (see the paragraphs describing transgenerational inheritance of DNA methylation and of chromatin states).

Evidence is now accumulating that provides insight in the mechanisms that underlie epigenetic transmission. These include the following: (1) DNA methylation, (2) histone modifications and chromatin remodeling, (3) inheritance of specific mRNAs, long non-coding RNAs (ncRNAs) and siRNAs/miRNAs, (4) feedback loops through which mRNA or protein products of a gene can stimulate its own transcription and enable “heritable states” of gene expression, and (5) the activity of chaperones such as Hsp90 that plays an important role in chromatin remodeling and can mediate epigenetic transgenerational variation. The most relevant mechanisms are described in Table 1.

**Table 1:** Molecular mechanisms of epigenetic transgenerational heredity

Steps	Molecular mechanisms
DNA sequence-invariant heritable traits	DNA methylation/histone post-translational modifications
DNA methylation maintenance across cell division cycles	Hemimethylated DNA-guided, DNMT1-mediated CpG methylation pattern maintenance
DNA demethylation	Passive DNA demethylation
	5-mC to 5-hmC conversion
	Active DNA demethylation
	Glycosylase-mediated base removal and base excision repair mechanisms
Histone code	Condensed chromatin
	HAT inactivation
	HMT activation
	Relaxed chromatin
	HAT activation
	HMT inactivation
Epigenetic modulation of mother-to-fetus transmission	Maternal nutrition status
	Maternal exposure to environmental toxins and food contaminants
	BPA
	Phthalates
	Dioxins
	Tobacco smoke
Cell differentiation and body development	Epigenetic signature reprogramming
	Erasure/reprogramming in the zygote (mitotic transmission)
	Erasure/reprogramming in PGCs (meiotic transmission)
	Gamete-carried transmission
	DNA methylation profiles in sperm and oocytes
	H3K4 and H3K27 histone methylation in sperm cells
	RNA molecules carried by sperm cells (mRNA, non-coding RNA, miRNA, siRNA, piRNA)
Stem cell reprogramming	Epigenetic signature of induced pluripotency
	Decreased TETs/decreased hydroxymethylation at ES gene promoters
	Reprogramming-resistant regions enriched for H3K9me3

### DNA methylation

**Fig. 1:**
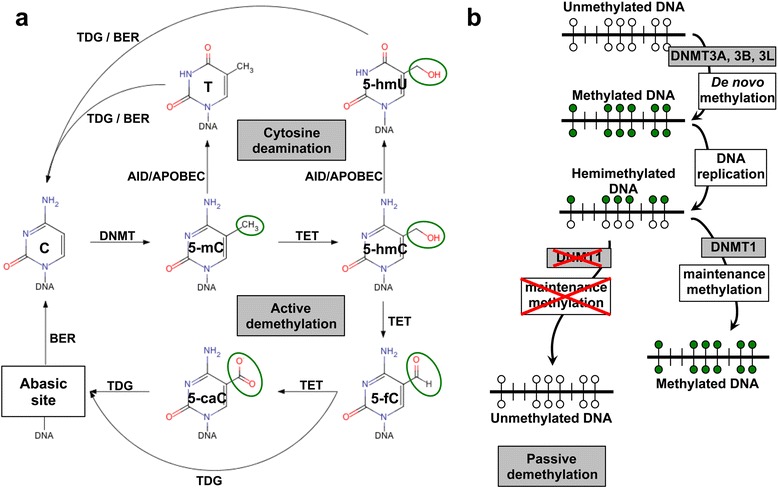
DNA methylation/demethylation mechanics. **a** Methyl groups (*green circles*) are transferred to C in order to generate 5-mC. DNA methyltransferases (DNMT) catalyze this process. In the “active DNA demethylation” the TET DNA demethylase converts 5-mC to 5-hmC, which is further processed to 5-fC and 5-caC. These residues are targets for the DNA repair pathway, whose most critical component is the hTDG, which is responsible also for the repair of U:G and T:G mismatches. DNA demethylation can also occur through spontaneous cytosine deamination, which is catalyzed by AID/APOBEC enzymes. This gives rise to 5-hmU and T bases. Transient U:G and T:G mismatches can be repaired by the TDG/BER pathway. **b** De novo and maintenance methylation occur using unmethylated DNA and hemimethylated/post-replication DNA as templates for DNMT enzymes. In the absence of maintenance methylation, progressive dilution of 5-mC or its oxidized derivatives at DNA replication can determine the appearance of unmethylated DNA. This process is known as “passive DNA demethylation”

Approximately 60–80 % of the 28 million CpG dinucleotides in the human genome are methylated [18, 19]. DNA methyltransferases (DNMT) recognize hemimethylated sites (maintenance methylation) or specific unmethylated sequences (de novo methylation) (Fig. 1). Maintenance DNA methylation occurs during DNA replication and is predominantly dependent on DNMT1, whereas de novo DNA methylation is carried out by DNMT3A, DNMT3B [20], and DNMT3L [21, 22]. Both DNMT3A and DNMT3B localize to methylated, CpG-dense regions and preferentially bind to the bodies of transcribed genes but are excluded from regions of active promoters and enhancers [23].

DNA methylation at CpG islands modulates gene transcription and is involved in alternative promoter usage and regulation of short and long ncRNA processing and of enhancer activity [24–26]. DNA methylation also affects determinants of higher-order DNA structure, e.g. in X chromosome inactivation, imprinting control regions (ICR) [27], heterochromatin folding and maintenance of genomic stability [28, 29].

### DNA demethylation

The dynamic on/off switching of gene expression requires a balanced action of DNA methylation versus DNA demethylation. Both active and passive DNA demethylation have indeed been shown to occur.

Active DNA demethylation, i.e. replication-independent enzymatic removal of 5-methyl cytosine (5-mC), occurs through the processing of 5-mC to 5-hydroxymethyl cytosine (5-hmC) catalyzed by the ten-eleven translocation (TET) dioxygenases [30–32]. 5-hmC is then converted to 5-formylcytosine (5-fC) and 5-carboxylcytosine (5-caC) [30]. The thymine DNA glycosylase (TDG) efficiently excises both 5-fC and 5-caC [33, 34]. This leaves an abasic site that is subsequently processed through the base excision repair (BER) pathway [33] (Fig. 1).

Passive DNA demethylation can also proceed through the processing of 5-mC to 5-hmC [30–32] (Fig. 1). Neither maintenance methylase DNMT1 nor de novo methylases DNMT3A and DNMT3B recognize 5-hmC, and no mechanisms for 5-hmC maintenance have been as yet identified [32]. Hence, 5-hmC is inevitably lost at each replication cycle [35]. This appears to have a functional impact on PGC, which progressively lose 5-mC between embryonic day (E) E9.5 and E10.5, until 5-mC becomes undetectable by E11.5 [31]. This progressive loss of 5-mC occurs concurrently with an enrichment of 5-hmC, suggesting a genome-scale conversion of 5-mC to 5-hmC for the epigenomic reprogramming of these cells [31].

An additional path of passive DNA demethylation proceeds through destabilization of DNMT1, which is then followed by “loss by dilution” of 5-mC through successive replication cycles. Overexpression of SET7 leads to decreased DNMT1 levels via induction of proteasome-mediated degradation [36]. SET7 directly interacts with DNMT1 and specifically monomethylates Lys-142 of DNMT1 [36]. On the opposite side, AKT1 phosphorylates DNMT1 at Ser143 and stabilizes the protein [37]. Phosphorylation of DNMT1 at Ser143 interferes with monomethylation of the nearby Lys142 [37], making these two modifications mutually exclusive. *Rb* and *ATM* also affect the stability of DNMT1 [38]. The inactivation of pRB promotes a Tip60 (acetyltransferase)-dependent ATM activation. This allows activated ATM to physically bind to DNMT1, forming a complex with Tip60 and the E3 ligase ubiquitin-like containing PHD and RING finger domain protein 1 (Uhrf1) and accelerates the DNMT1 ubiquitination driven by Tip60-dependent acetylation [38, 39]. In contrast, histone deacetylase 1 (HDAC1) and the deubiquitinase HAUSP (herpes virus-associated ubiquitin-specific protease) stabilize DNMT1 [39].

Of note, 5-mC frequently undergoes deamination (Fig. 1). Hence, a DNA methylation-dependent modification can end up in a permanently fixed DNA sequence change. Physiological enzymes are involved, raising the intriguing issue of “guided” mutagenesis of Mendelian traits. The AID and APOBEC enzyme families catalyze the cytosine processing which leads to cytosine deamination. This occurs predominantly on 5-hmC and 5-mC residues, giving rise to formation of 5-hydromethyluracil (5-hmU) and thymine (T) bases, respectively [40] (Fig. 1). Consequently, transient U:G and T:G mismatches can be generated, though most of these mutations can be efficiently repaired by the TDG/BER pathway [40]. Notably, dysregulated APOBEC3B-catalyzed deamination can provide a chronic source of DNA damage, with consequent TP53 inactivation; this was shown to lead to development of breast cancer [41].

### Reversible changes of epigenetic patterns

Epigenetic reprogramming through the mechanisms described above has been demonstrated in mammals over distinct, key developmental stages:

#### Erasure of DNA methylation patterns in the zygote

Erasure of DNA methylation patterns of the gametes (oocyte and sperm [42]) in the zygote was shown to occur immediately after fertilization. This process has been traditionally considered as a mechanism for resetting epigenetic marks between generations, to ensure the totipotency of the zygote after fertilization. Recent evidence from genome-scale DNA methylation analysis of human development confirmed a transient, highly dynamic state of global hypomethylation that affects most CpGs [43]. However, neither histone codes nor DNA methylation patterns are completely erased and are carried over through zygote divisions and generation of PGCs, thus, providing some of the means for transgenerational inheritance.

#### Erasure and reconstitution of DNA methylation patterns in PGCs

Epigenetic marks have been shown to undergo reprogramming across meiotic divisions of PGCs during gametogenesis. However, genome-wide DNA methylation profiling in PGCs revealed that, although the bulk of the genome becomes demethylated [44, 45], several loci escape this epigenetic erasure [46]. This leads to preserving the methylation status of more than 40 % of all 5-mC [16]. Substantial numbers of genes have been found to retain parental DNA methylation patterns in sperm and oocytes as a result of epigenetic transmission from PGCs [47]. It was recently reported that 5-hmC and 5-fC do exist in both maternal and paternal genomes and that 5-mC or its oxidized derivatives can be converted to unmodified cytosines through active demethylation rather than by passive dilution during embryonic development [48].

#### Erasure and reconstitution of epigenetic signatures during early body development

Reprogramming/re-establishment of epigenetic signatures was also shown to be necessary for proper development of a mature organism [49]. It has been shown that during early mitotic divisions of a mammalian embryo, daughter cells derived from the zygote have a globally hypomethylated genome [50] and a transcriptionally active chromatin due to histone H4 acetylation [51]. Therefore, they are an epigenetically homogeneous cell population [52, 53]. At the blastocyst stage, peripheral cells (that will become the extraembryonic tissue) have low levels of DNA methylation and are epigenetically different from cells of the inner cell mass (ICM, which will form the embryo) that have already undergone re-establishment of some methylation patterns [52, 53]. A major epigenetic switch then occurs during implantation at the transition from the blastocyst to the post-implantation epiblast [47].

#### Erasure and reconstitution of DNA methylation patterns in adult stem cells

Somatic cell nuclear transfer has been utilized as one of the procedures to obtain reprogramming of somatic cells toward a totipotent state, with the generation of induced pluripotent stem cells [54, 55]. This, and the possibility to isolate embryonic stem (ES) cells from the blastocyst ICM, provided unprecedented opportunities to investigate the mechanics of erasure and re-establishment of epigenetic patterns and to define the molecular components involved in these processes. Declining levels of TETs during differentiation were shown to be associated with decreased hydroxymethylation levels at the promoters of ES cell-specific genes [32]. Thus, the balance between hydroxymethylation and methylation in the genome appears to be linked with the balance between pluripotency and lineage commitment [32]. Moreover, reprogramming-resistant regions strongly enriched for H3K9me3 were demonstrated to be critical barriers for efficient reprogramming [56]. Hence, modulation of epigenetic inheritance appears to play a key role in stem cell differentiation/de-differentiation.

### Gamete-carried epigenetic traits

Nucleosomes are largely replaced by protamines in mature human sperm, thus erasing most chromatin patterns. However, not all histones in sperm are replaced by protamines, and epigenetic marks such as H3K4me2 and H3K27me3 have been detected in sperm [57, 58]. These retained nucleosomes are significantly enriched at loci of developmental importance, including imprinted gene clusters, microRNA clusters, HOX gene clusters, and the promoters of stand-alone developmental transcription and signalling factors [57]. H3K4me2 was found to be enriched at promoters of genes coding for developmental transcription factors, whereas H3K4me3 was found predominantly localized at promoters of genes important for spermatogenesis and rearrangement of nuclear architecture and presumably active during the gametogenesis [57].

Our findings indicated that sperm methylation pattern of the CD5/Leu1 and CD8/Leu2 genes is incompletely erased. This had a heavy impact on gene function and tightly prevented the expression of the CD5 gene, though not of CD8 [59]. Genes encoding olfactory receptors, in cases where mice associated specific odors with fearful experience, were also found differentially methylated in sperm, and this methylation pattern was transmitted to F1 and F2 generations [60].

RNA molecules packaged in sperm represent an additional contributor to transgenerational transmission of epigenetic traits and have been shown to profoundly affect offspring phenotypes [61, 62]. Injecting sperm RNA from traumatized males into fertilized wild type oocytes reproduced the altered behavior in the offspring. Moreover, miRNA-mediated signals can change DNA methylation patterns in the F2 sperm, and this signature can be maintained and replicated through subsequent mitotic and meiotic cycles [63].

Notably, RNA expressed in somatic cells can be transferred to gametes via extracellular vesicles [64]. Subcutaneous injection of human melanoma cells stably expressing enhanced green fluorescent protein (EGFP) led to the transfer of EGFP mRNA in murine sperm heads, likely through exosomes-mediated transport [65]. Furthermore, in *C. elegans*, it has recently been shown that neurons can transmit double-stranded RNA (dsRNA) to the germ cells to initiate transgenerational silencing of their target genes [16, 66]. Thus, extracellular vesicles have been revealed as an important route for transgenerational inheritance of epigenetic signatures.

### Transgenerational inheritance of DNA methylation patterns

Transgenerational inheritance of epigenetic patterns [16, 66] is key to a model of “epigenetic missing heredity”. In this regard, it should be noted that purely parental effects, such as the impact of direct in utero exposure to particular nutritional, hormonal, or stress/toxin environments, do not represent true transgenerational heredity [67]. In addition, F1 gametes are potentially exposed in utero to maternal experiences, and this may subsequently affect F2 offsprings. Hence, proof of transgenerational transmission of ancestral memory requires demonstrating the passing of the epigenetic trait through unexposed F2 gametes to F3 offsprings [16].

Physiological traits, such as body stature, were shown to rapidly and progressively adaptate to changing environments, e.g., nutritional status [2]. The epigenetic inactivation of height-associated genes (e.g., *BMP2*, *BMP6*, *CABLES1*, *DLEU7*, *GNAS*, *GNASAS*, *HHIP*, *MOS*, *PLAGL1*) was shown to be functionally equivalent to Mendelian physical loss of the corresponding alleles. Moreover, distinct epigenetic defects, such as in Beckwith–Wiedemann, Prader–Willi, Angelman’s, Rett, and Silver–Russell syndromes ([2] and references therein), were correspondingly shown to cause hereditary growth anomalies, indicating that body stature is under a heritable epigenetic control.

Correspondingly, the appearance of several pathological conditions with heritable components, such as diabetes and Crohn’s disease, is affected by interaction with dynamic environmental factors, such as host pathogens or nutritional status [68]. Exposure of parents to distinct diet regimens, stress, drugs, or endocrine/metabolic dysfunctions, was shown to additionally affect the transgenerational transmission of altered DNA methylation patterns [69–75]. As an example, F2 generation offspring (i.e., the grandchildren) of alcohol-abusing women have a higher tendency to show fetal alcohol syndrome than the F2 progeny of control women [76, 77].

Epigenetic modifications were also shown to be caused by exposure to environmental toxins, including metals (cadmium, arsenic, nickel, chromium, and methylmercury), solvents (trichloroethylene), air pollutants (black carbon, benzene), food-chain contaminants (dioxins), and tobacco smoke (nicotine, benzo(a)pyrene) [78, 79]. In utero and neonatal exposure to low doses of Bisphenol A and/or phthalates causes epigenetic alterations [71], such as differential methylation at CpG islands, histone modifications, and altered expression of ncRNAs and miRNAs [80, 81]. DNA methylation was shown to be affected by periconceptional maternal plasma concentrations of micronutrients involved in one-carbon metabolism, such as folate, B2 vitamin, methionine, and betaine [70]. The first evidence in mammals for a true transgenerational transmission of exposure-determined epigenetic traits was obtained in rats exposed to endocrine disruptors vinclozolin or methoxychlor during gestation. This induced in the F1 generation an adult phenotype of decreased spermatogenic capacity and of increased incidence of male infertility. Remarkably, transgenerational transmission of these effects through the male germ line was then observed in F1 to F4 generations [82]. DNMT3L has been reported to be necessary for maternal methylation imprinting [22]. DNMT3L is enzymatically inactive but acts as a stimulatory factor for de novo methylation by DNMT3A [21]. DNA methylation signatures driving altered behavioral/metabolic phenotypes (from exposure to maternal stress) were subsequently shown to be transgenerationally transmitted to the offspring [83], through miRNA delivery by sperm cells to the oocyte [63, 83].

### Transgenerational inheritance of chromatin states

Distinct types of histone post-translational modifications (PTMs) play a critical role in the nucleosome-dependent regulation of gene transcription. The largest body of knowledge has been gathered on histone methylation and acetylation [84], which cooperates in re-shaping chromatin organization. This histone code interacts closely with DNA methylation: unmethylated Lys-4 on H3 histone (H3K4) acts as a docking site for DNMT3A, which is recruited on nucleosomes and methylates associated target nucleotides [21, 22]. Recent findings showed that in the absence of histone H3, DNMT3A exists in an autoinhibitory form, in which the ATRX–DNMT3–DNMT3L (ADD) domain binds to the catalytic domain and hinders its DNA-binding capacity [85]. Once the DNMT3A–DNMT3L complex is recruited to the nucleosome, unmethylated H3K4 binds to the ADD domain and stimulates DNMT3A to undergo a significant conformational change from an autoinhibitory form to an active form that can bind DNA and exert DNA methylation activity [85]. Moreover, interaction of Dppa3 with histone H3K9me2 blocks the activity of TET3, favoring the maintenance of DNA methylation [86]. Less common histone PTMs have been recently identified [87, 88]. Among them, histone H1 arginine 54 citrullination (H1R54ci) determines histone displacement from chromatin and chromatin decondensation. Histone PTMs were further shown to be a critical mechanism for maintaining stem cell pluripotency [89].

**Fig. 2:**
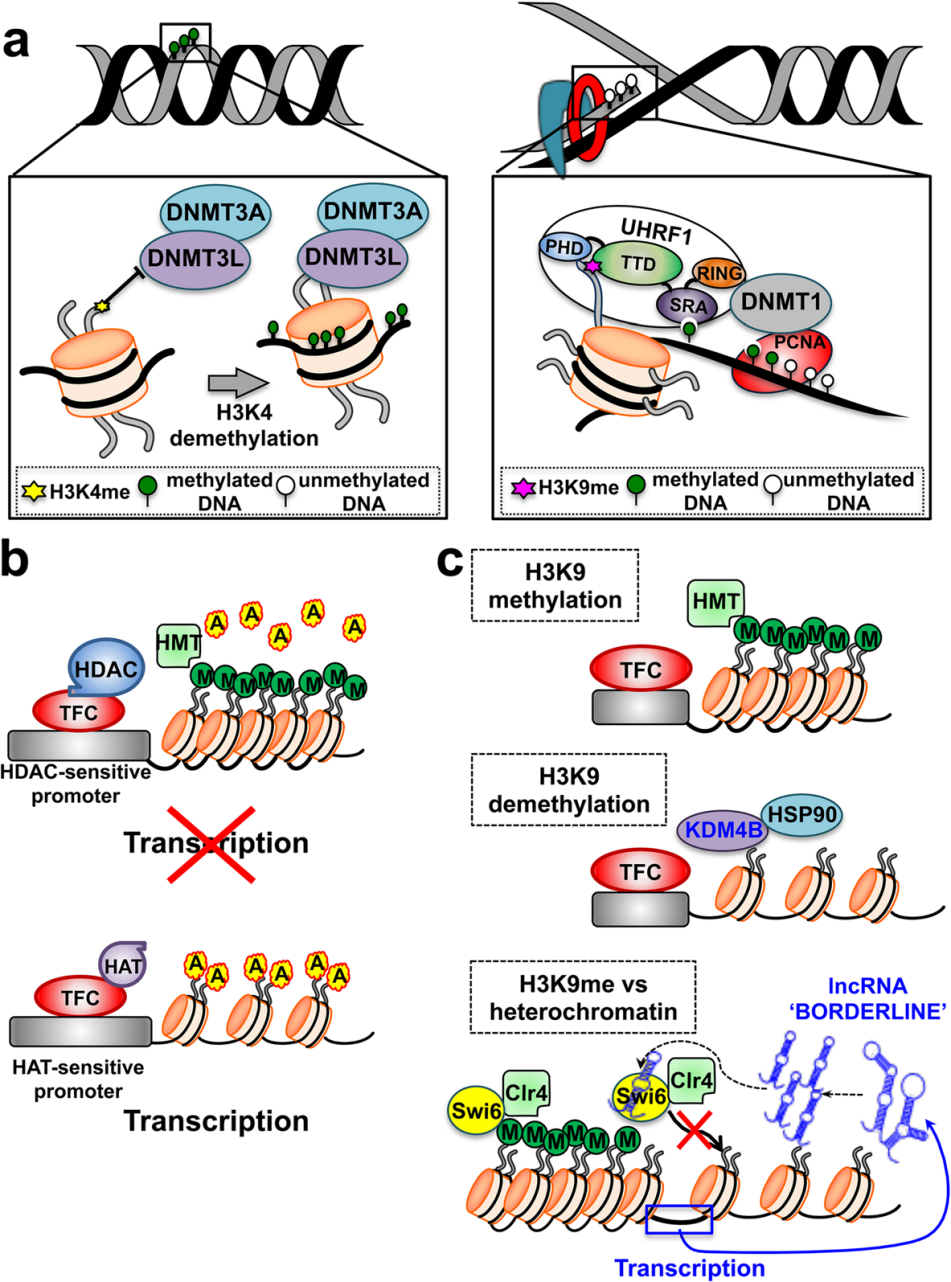
Histone modifications and DNA cooperate in re-shaping chromatin organization and regulating gene expression. **a** (*left*) De novo DNA methylation occurs on unmethylated DNA. It is catalyzed by the DNMT3, whose subunits can be positioned in proximity of their target sites through physical interaction with unmethylated H3K4. **a** (*right*) Maintenance DNA methylation occurs during the DNA replication and is catalyzed by the DNMT1. Uhrf1 and proliferating cell nuclear antigen (PCNA) associates to DNMT1 and recruit it to the replication fork, concentrating its activity on hemimethylated DNA. The Uhrf1 TTD domain interacts with H3K9me. This binding allows a faithful propagation of DNA methylation patterns throughout mitosis. **b** (*top*) HDAC and the transcription factor complex (TFC) can be recruited on sensitive promoters, leading to histone deacetylation. HMT-driven methylation of the histone tails causes tight wrapping of DNA around nucleosome cores and inhibition of gene expression. **b** (*bottom*) The accumulation of HAT-driven histone acetylation determines DNA relaxation around the nucleosomes surrounding HAT-sensitive promoters; this leads to increased transcription and gene expression. **c** Methylation of H3K9 plays a central role in non-DNA-dependent mechanisms of regulation of gene activity (*top*). Hsp90 has a strong effect on the histone code via stabilization of KDM4B, which demethylases H3K9 (*middle*). Non-coding RNAs alter the histone code through siRNA-dependent mechanisms that lead to direct competition between BORDERLINE ncRNAs and H3K9me for binding to the HP1 proteins, such as Swi6. This occurs at heterochromatin/euchromatin boundary sites and counteracts the spreading of heterochromatin into neighboring euchromatin (*bottom*). *A* acetyl groups, *M* methyl groups, *HAT* histone acetyltranferase, *HDAC* histone deacetylase, *HMT* histone methyltransferase

Histone PTMs and corresponding DNA methylation patterns can affect imprinting in mammalian cells [90, 91] through the selective recruitment of effector proteins, known as “readers” (e.g. the bromodomain motif that docks onto acetylated lysines [92]), which drive chromatin packaging around nucleosomes [88, 91] (Fig. 2).

Histone PTM-driven heritable silencing of gene expression is also affected by various chromatin remodeling factors. Among them, the polycomb-group (PcG) proteins recognize specific histone modifications such as the H3K27me3 and participate in maintenance of repressed chromatin domains [93]. PcG proteins take part to the regulation of X-chromosome inactivation and maintenance of stem cell identity [94]. Correspondingly, the molecular chaperone Hsp90 was shown to alter the histone code via interaction and stabilization of KDM4B, which demethylates H3K9. Pharmacological inhibition of Hsp90 results in ubiquitin-dependent proteasomal degradation of KDM4B, which is accompanied by increased methylation of H3K9 [95] (Fig. 2).

Although the mechanisms through which DNA methylation states can propagate across cell divisions have been studied in depth, it is still unclear how the histone code (e.g., the histone PTM levels) is restored through multiple rounds of DNA replication. During DNA replication, nucleosomes are disrupted ahead of the replication machinery and reassembled on the two newly synthesized DNA strands. Histones PTMs are transmitted with high efficiency at replication forks, indicating a specific recycling of old histones. However, incorporation of new, largely unmodified histones also occurs [96–98]. Reincorporated parental, modified histones may serve as a blueprint to modify neighboring new histones, limiting the possible “dilution” of the corresponding code. Recent findings suggested that most PTM levels are maintained according to the simple paradigm that new histones acquire modifications to become identical to the old ones [99]. These and other findings also demonstrated that H3K9me3 and H3K27me3 are central marks for cellular memory [97, 100], and are propagated by continuous modification of both new and old histones through the generations. Epigenetic heritability of H3K9 methylation was recently investigated in fission yeast and demonstrated to involve the activity of a single H3K9 methyltransferase, Clr4, that directs all H3K9 methylation and heterochromatin through a “read-write” mechanism [101, 102]. Hence, histones act as carriers of epigenetic information, and the kinetics of PTM restoration appears to play a critical role in epigenetic inheritance [90, 98].

### Epigenetic inheritance in model organisms

Non-DNA methylation-mediated epigenetic heritability has been demonstrated also in non-mammalian species, such as *C. elegans* [1], *D. melanogaster* [103], and *S. pombe* [104].

*C. elegans* lacks DNA methylation and has evolved histone methylation/demethylation pathways to regulate the transmission of epigenetic information through multiple generations [1, 105]. In *C. elegans*, transgenerational inheritance has been shown to be mediated by transmission of piRNAs. piRNAs induce a highly-stable, long-term gene silencing, which persists at least through 20 generations. The inheritance of the phenotype then becomes independent of the original piRNA, as is taken over by siRNAs [106]. These siRNAs act by modulating transcriptional gene silencing of histone methyltransferases, with consequent rearrangement of the chromatin structure [106]. Such heterochromatin-like configuration is required for stable silencing [16].

*D. melanogaster* shows “paramutations,” a form of epigenetic inheritance whereby one allele at a gene locus is capable of inducing a structural modification in the paired allele, in the absence of DNA sequence changes, which is then inherited through meiotic divisions [103]. The paramutated allele itself becomes paramutagenic and is capable to epigenetically convert a new paramutable allele. The paramutation has been shown to occur without any chromosome pairing between the paramutagenic and the paramutated loci and is mediated by maternal inheritance of piRNAs.

In the yeast *S. pombe*, transgenerational inheritance has been demonstrated to be mediated by multiple long ncRNAs termed BORDERLINEs, which act in a sequence-independent but locus-dependent manner [104] (Fig. 2). BORDERLINE ncRNAs are processed by Dicer into short RNAs referred to as brdrRNAs [104]. brdrRNAs then compete with H3K9me for binding to the HP1 protein Swi6. This prevents spreading of the HP1 protein Swi6 and histone H3K9 methylation beyond pericentromeric repeat regions and leads to Swi6 removal from chromatin, which counteracts the spreading of heterochromatin into neighboring euchromatin by preventing the spreading of H3K9me [104].

### Phenotypic impact of epigenetic heredity

Epigenetic alterations can have strong impact on hereditary disease phenotypes. Altered balance of epigenetic networks has been reported to cause major pathologies, including complex phenotype syndromes and cancer (Tables 2 and 3).

**Table 2:** Epigenetic hereditary traits contribution to developmental diseases^a^

Non-cancerous syndromes	Phenotypes/clinical features	Molecular defects
ATR-X	Upswept frontal hair line; hypertelorism; epicanthic folds; flat nasal bridge; small triangular upturned nose; tented upper lip; everted lower lip; hypotonic facies	Mutations in *ATRX* gene, hypomethylation of specific repeat and satellite sequences
Fragile X	Mild to severe intellectual disabilities; elongated face; large or protruding ears; macroorchidism; stereotypic movements (e.g., hand-flapping); social anxiety	Expansion and methylation of CGG repeat in *FMR1* 5′UTR, promoter methylation
ICF	Hypertelorism; low-set ears; epicanthal folds; macroglossia	*DNMT3B* mutations, DNA hypomethylation
Angelman	Severe intellectual and developmental disabilities; sleep disturbance; seizures; jerky movements (e.g., hand-flapping); frequent laughter or smiling; a happy behavior	Deregulation of one or more imprinted genes at 15q11–13 (maternal)
Prader–Willi	Low muscle tone; short stature; incomplete sexual development; cognitive disabilities; chronic feeling of hunger leading to excessive eating and life-threatening obesity	Deregulation of one or more imprinted genes at 15q11–13 (paternal)
Beckwith–Wiedemann	Macroglossia; macrosomia; midline abdominal wall defects; ear creases or ear pits; neonatal hypoglycemia	Deregulation of one or more imprinted genes at 11p15.5 (e.g., *IGF2*)
Rett	Small hands and feet; decelerated rate of head growth; repetitive stereotyped hand movements (e.g., wringing and/or repeatedly putting hands into the mouth); gastrointestinal disorders; seizures; no verbal skills; scoliosis; growth failure; constipation	*MeCP2* mutations
Rubinstein–Taybi	Short stature; moderate to severe learning difficulties; broad thumbs and first toes; increased risk of developing benign and malignant tumors, leukemia, and lymphoma	Mutation in CREB-binding protein (histone acetylation)
Coffin–Lowry	Abnormal growth; cardiac defects; kyphoscoliosis; auditory and visual abnormalities	Mutation in Rsk-2 (histone phosphorylation)
Silver–Russel	Feeding problems; hypoglycemia; excessive sweating; triangular shaped face with a small jaw and a pointed chin that tends to lessen slightly with age; curved down mouth; blue tinge to the whites of the eyes in younger children; normal size of head circumference, disproportionate to a small body size; wide and late-closing fontanelle; clinodactyly; body asymmetric growth; precocious puberty; low muscle tone; gastroesophageal reflux disease; lack of subcutaneous fat; late closing of the opening between the heart hemispheres; constipation	Loss of methylation on the *ICR1* paternal allele at the *H19*/*IGF2* locus (11p15)

^a^Phenotype-genotype correlations were extracted from the OMIM databank (www.ncbi.nlm.nih.gov/omim)

**Table 3:** Epigenetic heredity of cancer-causing genes

Cancers	
Bladder	Aberrant methylation of *TWIST*, *NID2*, and *RUNX3*
Brain	Aberrant methylation of *RASSF1A* and *MGMT*
Breast	Aberrant methylation of *BRCA1*, *Sat-2*, *IGF2*, *ATM*, *RASSF1A*, and other genes
Cervix	Hypermethylation of *CDKN2A*/*p16*
Colon-Rectum	Aberrant methylation of *MHL1*, *SEPT9*, *IGF2*, *THBD*, *C9orf50*, and other genes
Esophagus	Aberrant methylation of *CDH1*, *PIGR*, *RIN2*, and other genes
Head/Neck	Hypermethylation of *CDKN2A*/*p16* and *MGMT*
Kidney	Hypermethylation of *TIMP*-*3*
Leukemia	Hypermethylation of *p15* and chromosomal translocations involving HATs and HMTs
Liver	Aberrant methylation of multiple genes
Lung	Hypermethylation of *CDKN2A*/*p16*, *p73*, *RARb*, *RASSF1A*, *GSTP1*, *MGMT*, and other genes
Lymphoma/Myeloma	Hypermethylation of *DAPK*
Ovary	Hypermethylation of *BRCA1* and hypomethylation of *SAT2*
Pancreas	Hypermethylation of *APC* and hypomethylation of other genes
Prostate	Hypermethylation of *BRCA2*
Rhabdomyosarcoma	Hypermethylation of *PAX3*
Stomach	Hypomethylation of *Cyclin D2*
Thymus	Hypomethylation of *POMC*
Urothelial	Hypomethylation of Satellite DNA
Uterus	Hypermethylation of *hMLH1* leading to Microsatellite instability

Most hereditary diseases linked to defect of epigenetic control present with multi-organ abnormalities and overall developmental defects. Several of these syndromes are characterized by mental retardation and other central nervous system defects. Bone and cartilage growth abnormalities are also frequent, consistent with a key role of epigenetic regulation in body development [2, 3].

Notably, several epigenetic, hereditary syndromes are also characterized by gross chromosomal anomalies. This is consistent with the role that epigenetic mechanisms that play in the regulation of chromosome architecture and maintenance of genomic stability [28, 29], as well as in modulation of regulatory networks that involve p53.

Epigenetic changes can also have a major role in the development of cancer [107]. Most studied examples include patients with sporadic colorectal cancer with a microsatellite instability phenotype that shows methylation and silencing of the gene encoding MLH1 (Table 3), indicating that epigenetic silencing can result directly in genomic instability in transformed cells [108, 109]. However, epigenetic regulation of key oncogenes/tumor suppressor genes appears much more widespread than commonly appreciated, as major targets include cyclins, cyclin inhibitors, APC, BRCA1, retinoic acid receptors, protease modulators, IGF2, and transcription factors associated with epithelial-mesenchymal transition, e.g. Twist.

## Conclusions

**Fig. 3:**
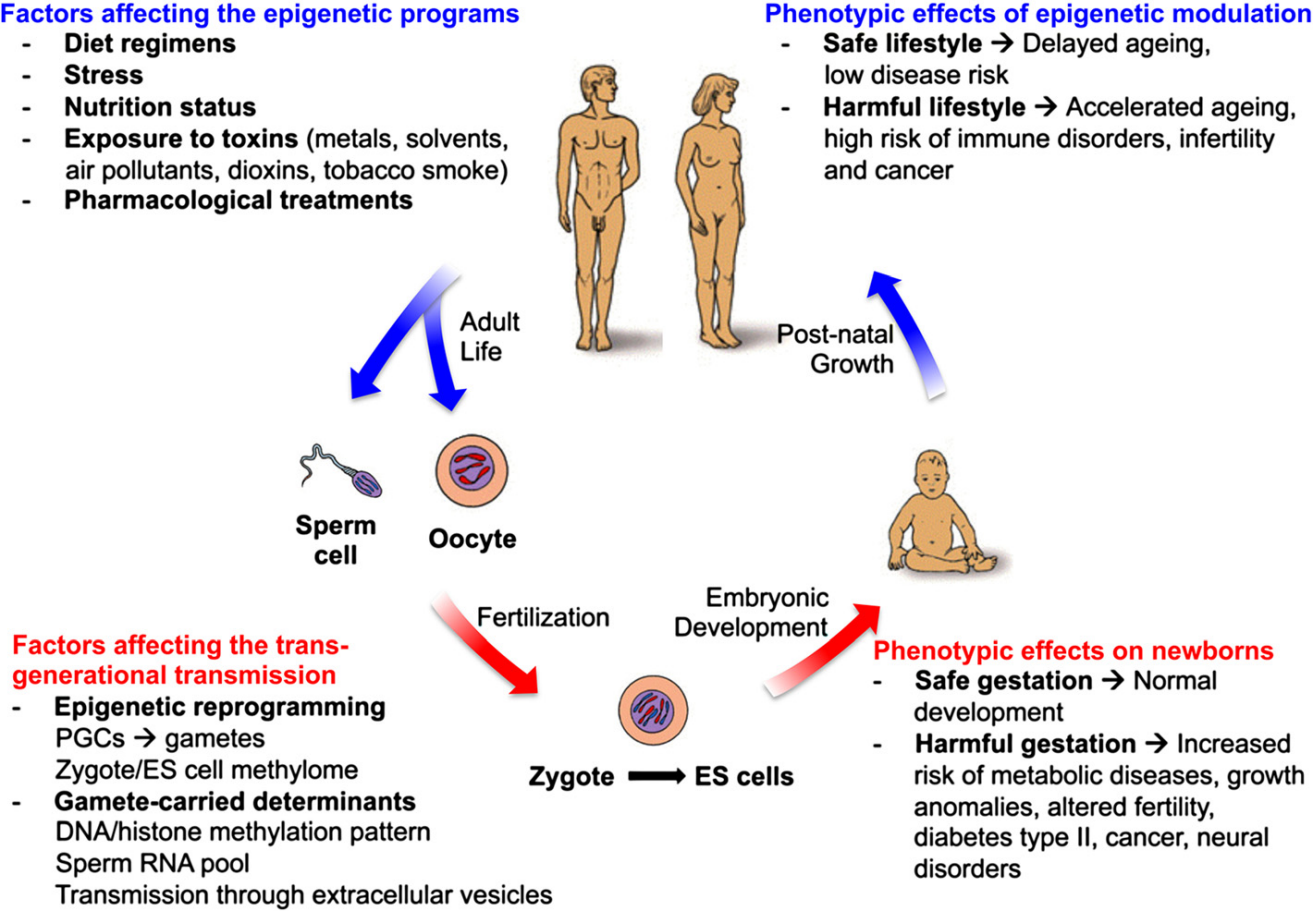
Epigenetic factors influencing human development and growth. The human life cycle is represented in the scheme. Major factors influencing the epigenetic programs and the maintenance of epigenetic patterns at both DNA and chromatin (histone code) levels are the maternal lifestyle during pregnancy and the personal exposure to harmful environments during post-natal growth and adult life

The genomes of eukaryotic organisms are adaptable to non-genetic/environmental-driven changes through epigenetic modulation of gene expression across generation cycles (Fig. 3). Epigenetic modifications include DNA methylation and histone modifications. These have the ability to alter gene expression patterns without affecting the nucleotide sequence of the underlying genome, the only exception being deamination of 5-mC to thymine. Tight regulation of the activity of DNA methyltransferases as well as of demethylases plays a mechanistic role in the establishment, maintenance and transient erasure of DNA methylation patterns. Correspondingly, post-translational modifications of histones and of regulatory proteins were shown to play a role in hereditary transmission of chromatin composition and configuration states, both in mammals and in model organisms. Thus, epigenetic programs contribute to the transgenerational inheritance of complex traits, which may help accounting for the “missing heritability” in current GWAS studies.

## Abbreviations

5-caC: 5-carboxylcytosine
5-fC: 5-formylcytosine
5-hmC: 5-hydroxymethyl cytosine
5-hmU: 5-hydromethyluracil
5-mC: 5-methyl cytosine
BER: base excision repair
DNMT: DNA methyltransferase
dsRNA: double-stranded RNA
EGFP: enhanced green fluorescent protein
ES: embryonic stem
GWAS: genome-wide association studies
HDAC: histone deacetylase
ICM: inner cell mass
ICR: imprinting control regions
ncRNA: non-coding RNA
PcG: polycomb-group
PGC: primordial germ cells
PTMs: post-translational modifications
T: thymine
TDG: thymine DNA glycosylase
TET: ten-eleven translocation
